# Blended Coronary Revascularization with Drug-Coated Balloon and Drug-Eluting Stent: A Narrative Review on Rationale, Clinical Evidence, and Future Perspectives

**DOI:** 10.3390/jcm14217576

**Published:** 2025-10-25

**Authors:** Filippo Luca Gurgoglione, Eman Murad, Marco Frazzetto, Bernardo Cortese

**Affiliations:** 1 DCB Academy, 20143 Milano, Italy; filippolucagurgoglione@gmail.com (F.L.G.); amynbs@gmail.com (E.M.); marcogiuseppe.frazzetto@uhhospitals.org (M.F.); 2 Mohammed Bin Khalifa Bin Salman Al Khalifa Specialist Cardiac Centre (MKCC), Road 4524, Block 945, Awali P.O. Box 101, Bahrain; 3 Coronary Center, University Hospitals Harrington Heart and Vascular Institute, Cleveland, OH 44106, USA; 4 Fondazione Ricerca e Innovazione Cardiovascolare, Via Ettore Ponti 49, 20143 Milano, Italy

**Keywords:** coronary artery disease, drug-coated balloon, diffuse coronary disease

## Abstract

Percutaneous coronary intervention (PCI) with drug-eluting stents (DESs) is the most used revascularization strategy in current clinical practice. However, this approach is still associated with a non-negligible risk of adverse events, including late and very late in-stent restenosis (ISR) and stent thrombosis, even with newer-generation DESs. Notably, long stents and the use of overlapping stents have been consistently identified as independent predictors of both ISR and stent thrombosis. Drug-coated balloons (DCBs) have emerged as a viable alternative to DESs. Initially evaluated in specific clinical settings, such as small-vessel disease and ISR, DCBs have demonstrated promising results in the treatment of more complex coronary lesions and higher-risk patient populations, including elderly, diabetics and those at high bleeding risk. Their main advantage lies in avoiding permanent implantation of metallic struts and polymer coatings, thereby preserving coronary vasomotor function and promoting positive vessel remodeling and late lumen enlargement. As a result, a hybrid or blended revascularization strategy combining DESs and DCBs has gained increasing interest, offering the potential to harness the complementary benefits of both DESs and DCBs, while minimizing stent overlap and total stent length. Some studies have explored this approach, particularly for the treatment of diffuse coronary artery disease and bifurcation lesions. This narrative review aims to outline the pathophysiological rationale underlying a blended DCB/DES approach and to summarize the currently available clinical evidence. Furthermore, we discuss future perspectives for optimizing the combination DCB and DES PCI in real-world practice.

## 1. Introduction

Percutaneous coronary intervention (PCI) has revolutionized the treatment landscape of obstructive coronary artery disease (CAD), offering a less invasive alternative to coronary artery bypass grafting with comparable outcomes, particularly in patients with low to intermediate anatomical complexity [1].

The introduction of new-generation drug-eluting stents (DESs), featuring thinner struts, enhanced radial strength and deliverability and more biocompatible polymer coatings, has significantly reduced the incidence of PCI failure compared to both bare-metal stents and first-generation DESs [2].

Despite these advancements, in-stent restenosis (ISR) and late stent thrombosis (ST) continue to occur, collectively accounting for approximately 10% of all PCI procedures [3]. These events are relatively common, with target lesion failure (TLF) occurring in approximately 11.9% of patients at 5 years, and their incidence tends to increase over time due to chronic inflammation and the prothrombotic state induced by the polymer and metallic struts of stents [4]. These events are driven not only by stent-related characteristics but also by patient comorbidities and procedural features, such as total stent length (TSL), stent diameter, and overlapping segments [5,6].

Drug-coated balloons (DCBs) are semi-compliant balloons, typically composed of polyurethane or nylon, coated with antiproliferative drugs via an excipient, that enables efficient and homogeneous drug delivery to the target lesion. By adhering to the “leave nothing behind” principle, DCBs avoid permanent metallic scaffolds, thereby preserving endothelial function and allowing for positive vascular remodeling and late lumen enlargement [7,8,9]. These biological advantages have translated into favorable clinical outcomes, particularly in ISR [10] and small vessel disease (SVD) [11,12], with growing evidence supporting their use in more complex lesion subsets [13,14,15,16,17,18].

Importantly, DESs and DCBs are not mutually exclusive PCI strategies. In recent years, blended coronary revascularization, namely the combined use of DCBs and DESs, has gained attention as a tailored approach to optimize long-term outcomes.

This narrative review aims to summarize the rationale, current clinical evidence, and future perspectives of this approach in contemporary interventional cardiology practice.

## 2. Paclitaxel- Versus Sirolimus-Coated Balloons: Pharmacokinetic Profiles and Clinical Evidence

Contemporary DCBs utilize two main antiproliferative agents that differ in their pharmacologic mechanisms. Paclitaxel disrupts microtubule formation, resulting in irreversible cell-cycle arrest and direct cytotoxic effects. In contrast, sirolimus acts as a cytostatic compound by inhibiting the mammalian target of rapamycin pathway, thereby suppressing smooth muscle cell proliferation. Although sirolimus is less lipophilic than paclitaxel, it offers a broader therapeutic margin [19].

From an angiographic standpoint, some studies have shown that paclitaxel-coated balloons (PCB) use is associated with greater late lumen enlargement [20,21], characterized by an increase in minimal lumen diameter and/or volume between post-PCI and follow-up [22]. However, other investigations have reported no significant differences between PCB and sirolimus-coated balloons (SCB [23,24,25]).

Overall, comparative studies have demonstrated similar efficacy between PCB and SCB across various clinical scenarios, including ISR [23,26,27] and de novo lesions [20,24,27,28].

## 3. Incidence and Predictors of PCI/DES Failure

While second-generation DESs have substantially improved long-term outcomes compared to earlier stent platforms [2], PCI failure remains a relevant clinical concern, largely due to the inherent limitations of permanent metallic implantation and its impact on vascular healing and function [29].

The pathophysiology of PCI failure is multifactorial (Table 1). A key contributor is delayed arterial healing, characterized by incomplete endothelialization, persistent fibrin deposition, and ongoing local inflammation and thrombogenicity [30]. In addition, exaggerated neointimal hyperplasia, often associated with a low density of contractile smooth muscle cells, drives progressive luminal narrowing [31]. Another significant mechanism is neoatherosclerosis, defined by early atherosclerotic development within the neointima, including foam cell accumulation, macrophage infiltration, and necrotic core formation [32]. These pathological processes culminate in ISR and ST, which may manifest with a broad spectrum of clinical presentations.

Robust evidence from a patient-level meta-analysis of six randomized controlled trials (RCTs), including 10.072 patients treated with second-generation DESs, reported a 5-year cumulative (TLF rate of 11.9%, comprising 3.7% cardiac death, 3.9% target-vessel myocardial infarction (MI), 6.9% target lesion revascularization (TLR), and 1.7% ST [4].

The risk of TLF results from a complex interplay of clinical, biological, mechanical, and procedural factors. Procedural predictors such as TSL, reference vessel diameter (RVD), and stent overlap are consistently associated with adverse outcomes. In the meta-analysis, each 1 mm decrease in RVD was associated with a 26% increase in TLF risk (hazard ratio (HR) 0.740; 95% CI: 0.558–0.982), while each 1 mm increase in TSL was linked to a 1% higher risk (HR 1.010; 95% CI: 1.005–1.016) [33]. These associations have been corroborated by multiple large studies. In the EXAMINATION-EXTEND trial (n = 1.489 STEMI patients), every 5 mm increase in stent length resulted in +7% TLF risk at 10 years, primarily driven by TLR [33]. Similarly, among patients with chronic coronary syndromes treated with everolimus-eluting stents, those in the highest TSL tertile had significantly elevated TLR rates at 3 years (HR 2.49 vs. middle tertile; HR 2.92 vs. lowest tertile) [34]. The GRAND-DES registry identified stent length > 40 mm as an independent predictor of both TLF (HR 1.88) and ST (HR 2.20) [35]. Likewise, in the WIN-DES pooled analysis, women receiving stents ≥ 36 mm had a 3-year major adverse cardiovascular events (MACE) rate of 19.6%, compared with 9.2% in those treated with shorter stents [36].

Stent diameter is another critical determinant. SVD, typically defined as a vessel diameter ≤ 2.5 mm, is linked to higher rates of ISR and ST, primarily due to an unfavorable metal-to-lumen ratio and altered hemodynamics [10]. SVD is more prevalent among patients with diabetes and chronic kidney disease, which further increase the risk of PCI failure [37]. In a large real-world registry (>17,000 patients treated with second-generation DES), stent diameters ≤ 2.5 mm were independently associated with higher 1-year MACE and TLR rates (10.5% and 6.5, respectively), versus 8.0% and 3.1% for stents > 3.5 mm [37].

Long-term data from the SYNTAXES trial reported a 10-year mortality rate of 28.5% in patients receiving stents < 3 mm, compared to 17.6% in those with larger diameters [38]. Similarly, in the BIONICS trial, SVD (≤2.5 mm) was associated with a nearly twofold increase in TLF (9.7% vs. 5.9%; HR 1.70; 95% CI: 1.22–2.37; *p* < 0.01) and a fivefold increase in ST (1.4% vs. 0.3%; HR 5.25; 95% CI: 1.47–18.8; *p* < 0.01) [39].

Stent overlap, which occurs in up to 30% of PCI procedures, is another relevant predictor of adverse events. Overlapping DESs are associated with greater vessel injury, increased local drug exposure, increased underexpansion and delayed endothelialization. A pooled analysis of the ISAR-TEST 4 and 5 trials found higher 10-year rates of TLR (23.7% vs. 16.3% *p* < 0.001) and MI (8.4% vs. 5.2% *p* < 0.001) in patients with overlapping DESs [40]. Similarly, O’Sullivan et al. reported a higher 3-year TLF rate in patients with overlapping stents (20.8%; adjusted HR 1.46; 95% CI: 1.03–2.09) compared to those with multiple non-overlapping stents, and an even greater risk compared to those treated with a single DES (18.8%; adjusted HR 1.74) [41].

## 4. Why Combine DCBs and DESs

**Figure 1:**
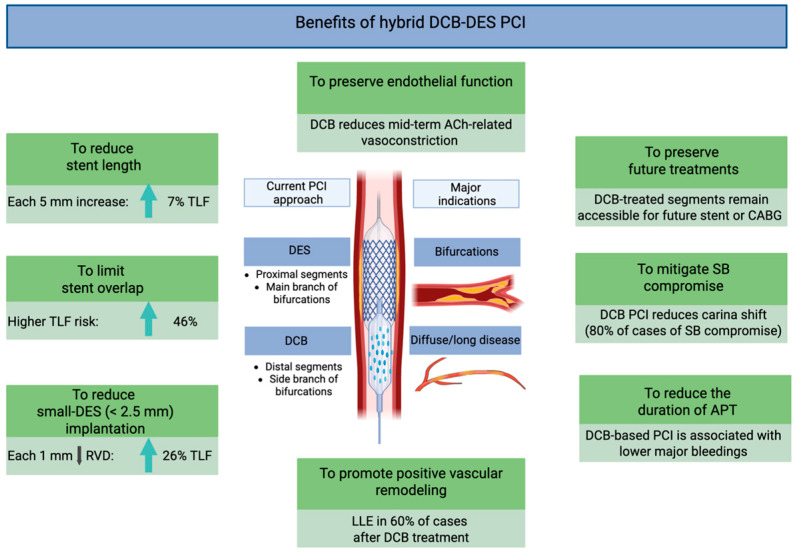
Mechanistic and clinical advantages of hybrid DCB/DES PCI. Abbreviations: ACh: acetylcholine; CABG: coronary artery bypass grafting; DCBs: drug-coated balloons; DESs: drug-eluting stents; LLE: late lumen enlargement; PCI: percutaneous coronary intervention; SB: side branch; TLF: target lesion failure.

The combined use of DCBs and DESs offers distinct mechanistic, procedural, and clinical advantages over conventional DES-only PCI (Figure 1). Rather than being viewed as mutually exclusive, DCBs and DESs should be considered complementary strategies, enabling a more tailored PCI approach based on specific patient characteristics and lesion-specific anatomical and physiological features.

### 4.1. Mechanistic Advantages

DCB therapy avoids the permanent implantation of metallic scaffolds and is associated with attenuation of the pathological responses typically induced by stent implantation [7,8]. Furthermore, DCBs allow for the preservation of endothelial vasomotor function, restoration of vascular physiology, and promotion of positive vessel remodeling.

A well-designed in vivo study by Kawai et al. assessed endothelial vasoreactivity 8 months after angioplasty using DCBs versus new-generation DESs. Following incremental intracoronary acetylcholine infusion, vasoconstrictive responses were significantly attenuated in the DCB group, suggesting superior preservation of endothelial function compared to DES-treated segments [42].

Moreover, DCB use has been associated with a higher incidence of late lumen enlargement, observed in up to 60% of cases [43]. This phenomenon is particularly notable with PCB, whose antiproliferative effect is mediated by inhibition of microtubule polymerization and activation of pro-apoptotic signaling pathways in vascular smooth muscle cells [19,22]. These pharmacodynamic effects are enhanced by optimal lesion preparation, especially in the presence of non-flow-limiting dissections, which enhance local drug uptake and diffusion within the vessel wall [44].

Additionally, DCBs provide homogeneous drug distribution across the treated segment, in contrast to DESs, which deliver the highest drug concentration near the stent struts and significantly less between the struts and at the stent edges [45].

### 4.2. Procedural Advantages

**Figure 2:**
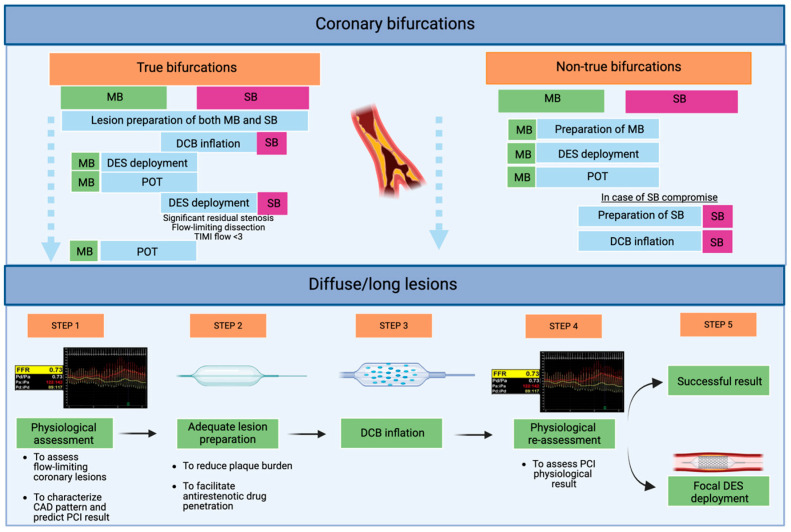
Application of hybrid DCB/DES PCI in current clinical practice: an exploratory proposal from our group. Abbreviations: DCBs: drug-coated balloons; DESs: drug-eluting stents; MB: main branch; PCI: percutaneous coronary intervention; POT: proximal optimization therapy; SB: side branch.

The hybrid DCB/DES strategy represents a personalized, patient-specific and lesion-specific approach to PCI, particularly valuable in the context of long, diffuse CAD and bifurcation lesions. DESs are typically reserved for segments more prone to recoil or requiring mechanical scaffolding. Conversely, DCBs may be optimally used in adjacent or downstream segments, with diffuse disease and small RVD (Figure 2).

This strategy mitigates several intrinsic limitations of DES-only PCI. By reducing total stent length and avoiding unnecessary stent overlap, the overall metallic burden is minimized. Furthermore, it helps preserve side branch (SB) patency, which is of prognostic relevance when supplying a significant myocardial territory, and avoids stent implantation in small vessels [7,8].

A frequently raised concern in early DCB studies was the occurrence of non-flow-limiting dissections following balloon angioplasty. However, a growing body of evidence now supports the safety of leaving such dissections untreated, provided that angiographic results are satisfactory and there is no evidence of compromised coronary flow [46]. Bailout stenting should be reserved for cases with suboptimal procedural outcomes or persistent flow limitation [47,48,49].

### 4.3. Clinical Advantages

Minimizing stent length may allow for shorter duration or de-escalation of dual antiplatelet therapy, a major contributor to bleeding risk, especially in elderly patients or those with a high bleeding risk profile [50]. Notably, recent studies have reported favorable outcomes following DCB angioplasty using shortened or stepwise antiplatelet therapy protocols [17,19,51]. Finally, an important advantage of DCB angioplasty is the preservation of future treatment options in non-stented vessel segments [52,53].

In summary, a major advantage of blended DCB/DES PCI lies in its “flexibility”, allowing the strategy to be tailored according to patient and lesion characteristics. For instance, in very young or elderly patients, a DCB-only approach may be preferred to minimize long-term stent-related complications and to reduce dual antiplatelet therapy duration, respectively. Conversely, in the presence of lesions prone to recoil or hemodynamically significant dissections, DES implantation should be favored, even when an initial DCB-only strategy is attempted. In all cases, careful and aggressive lesion preparation, particularly for complex coronary anatomy, remains the key determinant of success for an effective blended DCB/DES PCI strategy. This blended approach, however, requires an adequate learning curve to ensure proper lesion preparation and successful DCB delivery, ultimately improving long-term patient outcomes.

## 5. Research Methodology

### 5.1. Search Strategy and Selection Criteria

A systematic search of PubMed, Embase, and Scopus was conducted up to August 2025. The following search strategy was adopted “((hybrid percutaneous coronary intervention [Title/abstract] OR blended percutaneous coronary intervention [Title/abstract] OR combined drug coated balloon and drug eluting stent [Title/abstract]) AND (coronary artery disease [Title/abstract]))”.

### 5.2. Eligibility Criteria

Clinical studies were considered eligible if they: (i) enrolled patients undergoing PCI for obstructive coronary artery disease (CAD); (ii) employed blended DCB/DES PCI either as a single-arm intervention or in comparison with DES-only PCI; and (iii) reported post-discharge clinical outcomes. No restrictions were applied regarding PCI strategy, device platforms, eluting drugs, language of publication, or study design. Studies were excluded if they did not provide details on PCI procedural characteristics or lacked data on post-PCI outcomes.

## 6. Contemporary Clinical Evidence

### 6.1. Long Coronary Lesions

Costopoulos et al. conducted the first comparative matched-cohort analysis involving de novo long coronary lesions (>25 mm, mean length 47.3 mm). The DCB-based strategy (n = 93 lesions) included DCB-only PCI (56%), hybrid DCB/DES PCI (36.6%), and DCBs with bailout DESs (7.4%), compared against DES-only PCI (n = 93 lesions). DCBs were predominantly used in mid-to-distal vessel segments (95.7%) and were applied to vessels with significantly smaller RVD compared to DES angioplasty (2.44 ± 0.37 mm vs. 2.58 ± 0.29 mm; *p* < 0.01). Predilatation was not routinely performed (in 86% of cases). At 2-year follow-up, the incidence of MACE was comparable between the two groups (20.8% vs. 22.7%; *p* = 0.74), as were TLR rates (9.6% vs. 9.3%; *p* = 0.84) [52].

Xu et al. assessed 109 patients with de novo lesions > 25 mm and RVD between >2.0 and ≤2.75 mm. Compared with the Costopoulos cohort, a higher proportion of patients presented with acute coronary syndrome (ACS) (36.7% vs. 15.9%) and proximal lesions (43.0% vs. 4.3%). In 80.7% of cases, the preferred approach was proximal DES implantation (mean length 36.4 ± 19.3 mm), followed by distal DCB (mean length 25.3 ± 10.3 mm). Procedural success was 97.2%, with no in-hospital complications. At 19 months, MACE occurred in 6.4% of patients (TLR 2.8%; spontaneous MI 2.8%). Importantly, post-PCI quantitative flow ratio (QFR) was assessed in all patients. A QFR < 0.90 was associated with significantly higher MACE compared to QFR ≥ 0.90 (12.1 vs. 5.6; *p* = 0.035), highlighting the prognostic value of physiological assessment [54].

The REDUCE-STENT (dRug-coatED balloon angioplasty gUided by post-pErcutaneous coronary iNtervention pressure gradieNT) observational study evaluated a physiology Pd/Pa-guided approach in 109 patients with de novo lesions (mean lesion length: 36.3 ± 23.2 mm; mean RVD: 3.01 ± 0.51 mm), mainly with chronic coronary syndrome (CCS) (92%) and complex (ACC/AHA B2/C) lesion phenotype (94.5%). After lesion preparation, patients with Pd/Pa ≥ 0.90 underwent DCB-only PCI (79%). In cases of suboptimal physiological response, focal DESs were deployed, followed by repeat Pd/Pa measurement. If Pd/Pa was ≥0.90, a hybrid DCB/DES PCI strategy was adopted (14%), whereas DES-only PCI was reserved for persistently suboptimal result (6%). At 1-year follow-up, the rates of TLF and TLR were 13.2% and 8.7%, respectively [55].

In a subsequent study, the Pd/Pa-guided approach was applied to 100 de novo large lesions (RVD > 3.0 mm), of which 92.5% were long lesions. DCB-only PCI was used in 70% of cases (bailout DES was required in 6%), and blended DCB/DES PCI in 30%, typically for longer lesions (59.9 ± 22.5 mm vs. 38.1 ± 25.8 mm; *p* < 0.001). SCB were used in 77% of cases, PCB in 23%. At 12 months, TLF was 5.1%, numerically lower in the DCB-only group (1.5%) than in the hybrid group (10.7%; *p* = 0.073), with no deaths or target-vessel MI reported. These findings support using physiological guidance to safely reduce stent use while providing favorable clinical outcomes [56].

Gitto et al. compared DCB-based PCI (n = 147) with DES-only PCI (n = 701) for long de novo LAD lesions (>23 mm). Within the DCB group, 29.2% received DCB-only PCI, while 70.8% underwent hybrid PCI. In the latter, the DCB-treated segment exceeded the DES segment in 54.7% of cases (mean DCB-to-lesion length ratio 0.64 ± 0.29). After 1:1 propensity matching (n = 139 pairs), DCB-based PCI was associated with significantly lower 2-year TLF (3.5% vs. 18.2%; HR 0.20, 95% CI 0.07–0.58; *p* = 0.003), primarily driven by reduced TLR (3.5% vs. 14.6%; *p* = 0.011). Notably, most TLR events in the DCB group occurred within the first 6 months, with a plateau thereafter, contrasting with a continuous increase in TLR in the DES-only group, suggesting that long-term benefits of DCBs may become increasingly evident over time [57].

A large single-arm from Southeast Asia included 363 patients undergoing hybrid DCB/DES PCI for long lesions (81.0%) and bifurcation disease (19.0%). Most lesions (78%) were complex, and 99% of DCBs were coated with paclitaxel. The mean total treated length was 44.6 ± 23.3 mm (DES 28.7 ± 9.1 mm; DCB 25.5 ± 8.1 mm). No in-hospital TLF events occurred. At 1 year, TLF was 1.9%, with 0.6% cardiac death and 1.4% TLR (60% in DES-treated segments, 40% in DCB-treated segments) [58].

A recent retrospective study from South Korea compared 623 patients with de novo diffuse CAD involving lesions ≥ 30 mm who were treated with a DCB-based PCI strategy (of whom 73.7% underwent DCB-only angioplasty) with 623 propensity-matched patients from the large PTRG-DES registry (n = 13,160) who received second-generation DES-only PCI. The DCB-based approach was associated with a significantly shorter total DES length (8.1 ± 15.5 mm vs. 45.7 ± 15.9 mm; *p* < 0.001), a larger mean DES diameter (3.1 ± 0.5 mm vs. 2.8 ± 0.4 mm; *p* < 0.001), and a more than threefold lower use of small-diameter (<2.5 mm) DES (11.6% vs. 34.8%; *p* < 0.001). At 2-year follow-up, the DCB-based strategy was associated with a significantly lower incidence of MACE compared to DES-only PCI (4.6% vs. 14.6%; HR 0.29, 95% CI 0.18–0.47; *p* < 0.001). This difference was primarily driven by a lower rate of TVR (3.1% vs. 9.7%; *p* < 0.001) and major bleeding events (0.8% vs. 2.7%; *p* = 0.008). In multivariable analysis, DCB-based PCI remained independently associated with a significantly reduced risk of 2-year MACE, TVR, and major bleedings [59] (Table 2).

### 6.2. Bifurcation Lesions

Bifurcation lesions involve coronary segments adjacent to a significant SB that the operator aims to preserve during PCI. These lesions account for approximately 15–20% of all coronary revascularization procedures and are associated with increased periprocedural complications and worse clinical outcomes compared to non-bifurcation lesions [60]. Importantly, true bifurcation lesions, with significant disease in the SB, carry a markedly higher risk of cardiac death or MI compared to non-true bifurcations (HR 4.15) [61]. Several treatment strategies have been developed, including provisional stenting and complex two-stent techniques, each with specific indications. Current guidelines from the European Bifurcation Club recommend provisional stenting as the preferred initial approach [62].

DCBs offer unique mechanistic and procedural advantages in the bifurcation setting by minimizing metallic strut burden at the carina and reducing the risk of SB compromise due to carina shift [7,45,46]. Initial clinical experiences with DCBs in bifurcation PCI are mostly derived from single-arm studies, typically employing DCB angioplasty in the SB in conjunction with DES implantation in the MB (Table 3) (Figure 2).

The DEBSIDE trial enrolled 52 patients with bifurcation lesions and short SB lesions (≤6 mm lesion length, RVD 2.0–3.0 mm) treated with the Danubio PCB (Mynvasis, France, Danubio PCB, Mynvasis SAS, Lyon, France). Procedural success was 100%. At 6 months, late lumen loss (LLL) was –0.04 ± 0.34 mm, with only one MI (2%) reported [63].

Similarly, the BIOLUX-1 study evaluated the Pantera Lux DCB (Biotronik, Switzerland, Biotronik SE & Co. KG, Berlin, Germany) for SB treatment in 35 patients, reporting a mean LLL of 0.10 ± 0.43 mm, no restenosis, one TLR, and one possible target vessel MI [64].

The SARPEDON study enrolled 58 patients with SB diameter ≥ 2.0 mm, the majority of whom had Medina 1.1.0 (51.7%), followed by true bifurcation lesions (41.2%). A provisional strategy was employed, with Pantera Lux PCB delivered in the SB after DES implantation in the MB and kissing balloon inflation (KBI). Restenosis occurred in 6.0% of patients (all at the SB ostium), and the 12-month MACE rate was 19.0%, including three revascularizations [65].

The HYPER study (HYbrid APproach Evaluating a DCB + DES strategy in diffuse disease) included 50 patients with de novo true bifurcations (Medina 1.0.1, 0.1.1, or 1.1.1) and SB RVD 2.0–2.75 mm. Treatment involved second-generation DESs in the MB and Restore PCB (Cardionovum, Germany) in the SB. In 84% of cases, the DCB was inflated after MB stenting, with KBI in 95% of cases and proximal optimization technique (POT) in 5%. Procedural success was 96%, with no flow-limiting dissections or need for bailout stenting. At 1 year, one TLR occurred in the DES-treated segments, with no events in the SB [66,67].

Kasbaoui et al. evaluated 45 patients with true bifurcations (55.6% Medina 0,1,1 and 44.4% Medina 1,1,1), using the Agent PCB (Boston Scientific, Marlborough, MA, USA) in the SB. Compared to the HYPER cohort, these patients had a higher prevalence of ACS (53.2% vs. 8.0%) and larger SB diameter (RVD 2.55 ± 0.3 mm vs. 2.3 ± 0.5 mm). Notably, DCBs were deployed before MB stenting. Additional SB interventions, including re-POT or KBI, were required in 60% of patients. Bailout stenting of the SB occurred in 11.1% of cases (60% due to dissection, 40% due to residual stenosis). At 6 months, angiographic restenosis was observed in only 2.2%. Ostial SB lesion length > 10 mm was the only independent predictor of clinical failure (odds ratio 12.49; 95% CI: 1.17–133.6; *p* = 0.037) [68].

A German RCT enrolled patients with non-left main (LM) bifurcations and SB lesions < 10 mm (diameter: 2.0–3.5 mm). Following MB DES implantation, patients were randomized to either SeQuent Please PCB or POBA for SB treatment. At 9 months, the PCB group showed significantly reduced restenosis (6% vs. 26%; *p* = 0.045) and LLL (0.13 mm vs. 0.51 mm; *p* = 0.013) [69].

In the Chinese BEYOND trial, 222 patients with bifurcation lesions were randomized to receive the Bingo PCB (Yinyi Biotech, China) or POBA in the SB after MB stenting. At 9 months, the DCB group had significantly lower residual stenosis (28.7% ± 18.7% vs. 40.0% ± 19.0%; *p* < 0.0001), reduced LLL (–0.06 ± 0.32 mm vs. 0.18 ± 0.34 mm; *p* < 0.0001), and numerically lower major adverse cardiac and cerebrovascular events (0.9% vs. 3.7%; *p* = 0.16) [70].

The large DCB-BIF RCT randomized 784 patients with true bifurcation lesions and severely compromised SB to receive either DCB (n = 391) or non-compliant balloon (NCB; n = 393) following MB DES implantation. At 12 months, the DCB group had a significantly lower MACE rate (7.2% vs. 12.5%; HR: 0.56; 95% CI: 0.35–0.88; *p* = 0.013), largely driven by reduced MI rates [71].

**Table 3:** Most relevance studies on hybrid DCB/DES PCI for bifurcation lesions.

First Author, Date, Reference	Study Design	Study Population	DCB-Based Group	DCB Used	Main Results
Berland, 2015 [63]	Prospective, non-randomised, multicenter, study	52 patients with bifurcations and short SB (≤6 mm, RVD 2.0–3.0 mm)	PCB for SB after MB stenting	Danubio PCB (Mynvasis, France).	At 6 months, LLL was –0.04 ± 0.34 mm, with only one MI (2%) reported
Worthley, 2015 [64]	Prospective, multi-center, single arm pilot study	35 patients with bifurcations	SB: PCB MB: DES	Pantera Lux PCB (Biotronik, Switzerland)	Mean LLL of 0.10 ± 0.43 mm, no restenosis, one TLR
Jim, 2015 [65]	Observational study	58 patients with SB diameter ≥ 2.0 mm	PCB for SB after MB stenting and KBI	Pantera Lux PCB (Biotronik, Switzerland)	Restenosis occurred in 6.0% of patients (all at the SB ostium). 12-month MACE rate 19.0%.
Pellegrini, 2023 [66]	Sub-study of the prospective, single-arm, multicenter HYPER trial	50 patients with true coronary bifurcations	DCB inflated after MB stenting (84%), with KBI in 95% of cases	Restore (Cardionovum GmbH, Germany)	Procedural success was 96%. At 1 year, one TLR occurred in the DES-treated segments, with no events in the SB
Kasbaoui, 2023 [68]	Prospective, non-randomized, single-center study	45 patients with de novo true bifurcations	DCB deployed before MB stenting. Additional SB interventions required in 60%.	Agent PCB (Boston Scientific, USA	At 6 months, angiographic restenosis was observed in only 2.2%. Ostial SB lesion length > 10 mm was the only independent predictor of clinical failure
Kleber, 2016 [69]	RCT	DCB vs. POBA in 64 patients with non-LM bifurcations and SB lesions < 10 mm	DCB inflated in the SB after MB stenting	SeQuent Please PCB (B. Braun, Germany)	At 9 months, the PCB group showed reduced restenosis (6% vs. 26%; *p* = 0.045) and LLL (0.13 mm vs. 0.51 mm; *p* = 0.013
Jing, 2019 [70]	RCT	DCB vs. POBA in 222 patients with non-LM bifurcations	DCB inflated in the SB after MB stenting and KBI	Bingo PCB (Yinyi, China)	At 9 months, the DCB group had significantly lower residual stenosis (28.7 vs. 40.0%, *p* < 0.0001), reduced LLL (–0.06 mm vs. 0.18 mm; *p* < 0.0001).
Gao, 2025 [71]	RCT	DCB vs. non-compliant balloons in true bifurcations with SB stenosis ≥ 70%	DCB inflated in the SB after MB stenting and KBI	-	At 12 months, the DCB group had a significantly lower MACE rate (7.2% vs. 12.5%, *p* = 0.013), largely driven by reduced MI rates.

Table legend. DCB: drug-coated balloon; DES: drug-eluting stent; KBI: kissing balloon inflation; LLL: late lumen loss; LM: left main; MB: main branch; MACE: major adverse cardiovascular events; MI: myocardial infarction; PCB: paclitaxel-coated balloon; PCI: percutaneous coronary intervention; POBA: plain old balloon angioplasty; RCT: randomized controlled trial; SB: side branch; TLR: target lesion revascularization; RVD: reference vessel diameter.

### 6.3. Chronic Total Occlusion

The use of a DCB–DES blended PCI strategy significantly reduced the total length of implanted DES compared with DES-only PCI and may help avoid the “full-metal jacket” technique [72,73]. A meta-analysis from our group including five observational studies with 2.087 patients (mean follow-up 32 months) showed a trend toward lower rates of target vessel failure (0.45% vs. 0.76% person-months; *p* = 0.09) and cardiac death (0.05% vs. 0.10% person-months; *p* = 0.09) [15].

In a recent observational study of 291 patients with revascularized CTO and diffuse coronary disease, DES plus DCB PCI (n = 100) was compared with DES-only PCI (n = 191). At 24 months, the DES plus DCB group had significantly lower rates of MACE (26.0% vs. 41.4%; *p* = 0.008) and cardiovascular hospitalization (20.0% vs. 36.7%; *p* = 0.005) [74].

The ongoing PICCOLETO X multicenter retrospective study is further evaluating DCBs in CTO, including both de novo lesions and ISR, with 12-month MACE as the primary endpoint.

## 7. Future Perspectives

These promising results should be interpreted with caution for several reasons. First, most of the available studies are retrospective, and in some cases, the comparison with DES-based PCI was conducted using propensity-matched analyses. Furthermore, PCI strategies, including device selection, use of intracoronary imaging, and functional assessment, were left to operator discretion, introducing potential variability. Finally, studies with longer follow-up are needed to capture the potential long-term benefits of blended PCI strategies.

Moreover, several unresolved questions remain regarding the blended use of DCBs and DESs in coronary revascularizations.

### 7.1. Diffuse/Long Coronary Lesions

Current evidence is largely derived from small, observational, and often single-center studies.

The ongoing HYPER II trial (NCT05650450) aims to address these gaps. This prospective, non-randomized, multicenter clinical study aims to enroll up to 500 patients across 15 international centers. Eligible patients include those with stable or unstable CAD presenting diffuse de novo lesions exceeding 38 mm in length. The hybrid strategy involves the implantation of a new-generation DES in the proximal segment (RVD > 2.75 mm) of the target vessel, followed by DCB inflation in the distal segment (RVD 2.0–2.75 mm). This study employs the Restore PCB, with the primary endpoint being TLF at 12 months.

Furthermore, although initial studies have documented favorable outcomes following DCB-based PCI in multivessel CAD [75,76], particularly among high-risk subsets such as patients with diabetes [77], the efficacy of a blended DCB/DES approach still requires validation in larger, adequately powered RCTs.

A key unresolved issue pertains to the role of intracoronary imaging in guiding hybrid PCI. Previous studies reported relatively low use of intracoronary imaging, ranging from 14.0% [54] to 39.8% [52]. Whether systematic implementation of optical coherence tomography or intravascular ultrasound could improve lesion selection, optimize stent and balloon deployment, or predict TLR remains an open question. Furthermore, it is unknown whether the efficacy of this approach varies according to the underlying plaque morphology, such as fibrotic, lipid-rich, or heavily calcified lesions or according to gender [78].

Another area of ongoing research involves the use of physiology-guided PCI to optimize or complement the DCB/DES strategy. Preliminary evidence suggests that invasive physiological assessment may help reduce unnecessary stenting and minimize metallic burden [50,51,52]. However, whether this translates into improved long-term clinical outcomes over DES-only PCI remains uncertain. Moreover, non-invasive angiography-derived physiological indices and tools like the Pullback Pressure Gradient, which differentiate focal from diffuse disease [79], are yet to be validated in this setting.

Emerging observations suggest that adverse events may plateau in DCB-treated segments, while continuing to accrue in DES-treated segments over time. This phenomenon highlights the need for long-term follow-up studies to better determine the efficacy and safety of hybrid PCI relative to conventional DES-only strategies.

### 7.2. Bifurcation Lesions

Current evidence in the setting of bifurcation lesions has established the superiority of DCB angioplasty over POBA, both in terms of angiographic outcomes and clinical endpoints [68,69,70,71]. Future lines of investigation are evolving in two distinct directions.

The first involves two ongoing RCT directly comparing the clinical efficacy of DCBs versus DESs for SB treatment in true bifurcation lesions.

The Hybrid DEB is a multicenter RCT enrolling 500 patients with de novo true coronary bifurcation lesions, presenting with either CCS or ACS. The trial commenced in 2023 and is expected to be completed by 2030. Eligible lesions must have significant SB stenosis (≥50%) or functionally significant intermediate stenosis (FFR ≤ 0.80 or iFR ≤ 0.89), and a reference SB diameter ≥ 2.5 mm. Patients will be randomized to either a hybrid strategy, involving DCB inflation in the SB (Magic Touch SCB, Concept Medical, Tampa, FL, USA), following MB stenting with the Supraflex sirolimus-eluting stent (Sahajanand Medical Technologies, Surat, India), or a planned two-stent approach using Culotte, TAP, or T-stenting techniques based on operator preference and lesion characteristics. The primary endpoint is a composite of all-cause death, periprocedural or spontaneous MI, and/or TVR at 2-year follow-up [80].

Similarly, the PROVISION-DEB trial is enrolling 750 patients with non-LM true bifurcation lesions undergoing PCI for either CCS or ACS. The study began in 2023 and is expected to conclude in 2028. Eligible SB must have a visually assessed diameter between 2.25 and 2.75 mm, with diameter stenosis ≥ 70% and lesion length ≥ 10 mm. Patients will be randomized 1:1 to either a provisional one-stent plus DCB strategy, with SB treatment timing (before or after MB stenting) left to the operator’s discretion, or a planned two-stent strategy using techniques such as Mini-Crush, DK-Crush, Culotte, TAP, or T-stenting. The primary endpoint is TLF at 3 years [81].

## 8. Conclusions

Blended DCB/DES PCI represents a feasible and promising revascularization strategy for patients with diffuse or long CAD and bifurcation lesions. Large observational studies have demonstrated its potential to reduce adverse events compared to traditional DES-only PCI. This approach should be seen as a step toward tailoring PCI strategies to individual patient and lesion characteristics.

Further RCT are warranted to validate these encouraging findings, define the optimal implantation technique, and clarify the roles of intracoronary imaging and physiological assessment in guiding blended DCB/DES PCI.

## Figures and Tables

**Table 1:** Appraisal of risk factors and mechanisms of in-stent restenosis.

Classification	ISR Risk Factor	Mechanism
Patient-related	Diabetes mellitus	Chronic hyperglycemia promotes smooth-muscle cell proliferation and extracellular-matrix deposition, causing exaggerated neointimal hyperplasia and delayed endothelial healing.
	CKD	Uremic toxins and oxidative stress induce endothelial dysfunction, chronic inflammation, and vascular calcification, leading to accelerated intimal thickening.
	Elderly age	Impaired vascular repair, oxidative injury, and higher prevalence of comorbidities promote neoatherosclerosis and delayed healing.
	Smoking	Pro-Inflammatory state accelerates intimal growth
Lesion-related	Long lesion length	Extensive arterial injury from long plaques increases smooth-muscle proliferation and inflammation
	Small vessel diameter (≤2.75 mm)	High metal-to-lumen ratio and disturbed flow amplify local inflammation and LLL.
	Diffuse coronary disease	Multiple long stents amplify the metallic burden, predisposing to neointimal proliferation.
	Heavy calcification	Prevents full stent expansion and even drug diffusion and promotes stent underexpansion and malapposition.
Procedure-related	Overlapping stents	Double metallic struts and polymer layers intensify vascular injury, delay endothelialization, and heighten inflammatory response.
	Stent under-expansion	Residual narrowing and poor apposition disturb flow, hinder drug delivery, and foster tissue growth.
	Stent malapposition	Malapposed struts maintain pro-thrombotic and pro-inflammatory microenvironments, delaying arterial healing.
	Stent fracture	Mechanical discontinuity and local vessel trauma promote recoil and focal restenosis at the fracture site.
Biological mechanisms	Delayed arterial healing	Incomplete endothelialization and persistent fibrin cause chronic inflammation and impaired vascular recovery.
	Neointimal hyperplasia	Proliferation and migration of smooth-muscle cells with extracellular-matrix promote progressive lumen narrowing.
	Neoatherosclerosis	Exaggerated formation of lipid-laden macrophages and necrotic core within neointima.
	Persistent fibrin & thrombogenicity	Fibrin deposition sustains platelet activation and long-term inflammation.
	Negative vascular remodeling	Chronic inflammation induces negative remodeling and LLL.

Table legend. CKD: chronic kidney disease; ISR: in-stent restenosis; LLL: late lumen loss.

**Table 2:** Current evidence on hybrid DCB/DES PCI for diffuse/long CAD.

First Author, Date, Reference,	Study Design	Study Population	DCB-Based Group	DCB Used	Main Results
Costopoulos, 2013 [52]	Observational, matched-based study	186 coronary lesions > 25 mm	56%: DCB-only PCI 36.6%: hybrid PCI 7.4%: DCB with bailout DES	87.1%: IN.PACT Falcon (Medtronic Inc., California) 12.9%: Pantera Lux (Biotronik, Germany)	The incidence of MACE was comparable between DCB-based PCI and DES-only PCI (20.8% vs. 22.7%; *p* = 0.74), as were TLR rates (9.6% vs. 9.3%; *p* = 0.84)
Xu, 2023 [54]	Observational case series study	109 pts (114 lesions) > 25 mm, RVD 2-2.75 mm	80.7%: DES-proximal, DCB-distal 19.3%: DCB-Proximal, DES-distal	58,3% SeQuent Please PCB (B. Braun, Germany) 40% Bingo PCB (Yinyi, China) 1.7% Restore PCB (Cardionovum, Germany)	At 19 months, MACE occurred in 6.4% of patients (TLR 2.8%; spontaneous MI 2.8%)
Leone, 2023 [55]	Retrospective observational study	109 lesions (mean length 36 mm), 94.5% complex	79%: DCB-only PCI 14%: hybrid PCI 6%: DES-only PCI	SCB: 87% PCB: 13%	At 1-year follow-up, the rates of TLF and TLR were 13.2% and 8.7%, respectively
Leone, 2023 [56]	Retrospective observational study	100 de novo large lesions (RVD > 3 mm), 92.5% long lesions	70%: DCB-only PCI 30%: hybrid PCI	SCB: 77% PCB: 23%	At 12 months, TLF was 5.1%, numerically lower in the DCB-only group (1.5%) than in the hybrid group (10.7%; *p* = 0.073)
Gitto, 2023 [57]	Retrospective, observational, matched-based study	139 matched pairs, with long de novo LAD lesions (>23 mm)	70.8%: hybrid PCI 29.2%: DCB-only PCI	SCB: 84% PCB: 14% Both: 2%	DCB-based PCI was associated with significantly lower 2-year TLF (3.5% vs. 18.2%, *p* = 0.003).
Teo, 2024 [58]	Retrospective, single-center observational study	363 patients with long lesions (81.0%) and bifurcations (19.0%)	100% hybrid PCI	99% PCB 1% SCB	At 1 year, TLF was 1.9%, with 0.6% cardiac death and 1.4% TLR
Shin, 2025 [59]	Retrospective, matched-based study	1246 patients with de novo CAD lesions ≥ 30 mm	73.7% DCB-only PCI 26.3% hybrid PCI	SeQuent Please PCB (B. Braun, Germany)	At 2-year, DCB-based PCI was associated with a lower incidence of MACE compared to DES-only PCI (4.6% vs. 14.6%, *p* < 0.001).

Table legend. CAD: coronary artery disease; DCB: drug-coated balloon; DES: drug-eluting stent; LAD: left anterior descending; MACE: major adverse cardiovascular events; PCB: paclitaxel-coated balloon; PCI: percutaneous coronary intervention; RVD: reference vessel diameter; SCB: sirolimus-coated balloon; TLF: target lesion failure; TLR: target lesion revascularization.

## Data Availability

Not applicable.

## Abbreviations

The following abbreviations are used in this manuscript:

ACS	acute coronary syndrome
CAD	coronary artery disease
CCS	chronic coronary syndrome
DCB	drug-coated balloon
DES	drug-eluting stent
HR	hazard ratio
ISR	in-stent restenosis
KBI	kissing balloon inflation
LLL	late lumen loss
MACE	major adverse cardiovascular events
MB	main branch
MI	myocardial infarction
LM	left main
PCB	paclitaxel-coated balloon
PCI	percutaneous coronary intervention
POBA	plain old balloon angioplasty
POT	proximal optimization therapy
QFR	quantitative flow ratio
RCT	randomized controlled trial
RVD	reference vessel diameter
SB	side branch
SCB	sirolimus-coated balloon
ST	stent thrombosis
